# Quantitative Analysis of Cell Aggregation Dynamics Identifies HDAC Inhibitors as Potential Regulators of Cancer Cell Clustering

**DOI:** 10.3390/cancers13225840

**Published:** 2021-11-21

**Authors:** Fabien Gava, Julie Pignolet, Sébastien Déjean, Odile Mondésert, Renaud Morin, Joseph Agossa, Bernard Ducommun, Valérie Lobjois

**Affiliations:** 1 Institut des Technologies Avancées en Sciences du Vivant (ITAV)-USR3505, Université de Toulouse, CNRS, Université Paul Sabatier, 31100 Toulouse, France; fabien.gava@inserm.fr (F.G.); julie.pignolet@univ-tlse3.fr (J.P.); odile.mondesert@cnrs.fr (O.M.); josephagossa@yahoo.fr (J.A.); bernard.ducommun@univ-tlse3.fr (B.D.); 2 Institut de Mathématiques de Toulouse (IMT)-UMR5219, Université de Toulouse, CNRS, Université Paul Sabatier, 31062 Toulouse, France; sebastien.dejean@math.univ-toulouse.fr; 3 Imactiv-3D SAS, 1 Place Pierre POTIER, 31100 Toulouse, France; renaud.morin@imactiv-3d.com; 4 CHU de Toulouse, 31000 Toulouse, France; 5 Molecular, Cellular & Developmental Biology Unit (MCD)–UMR5577, Center for Integrative Biology (CBI), Université de Toulouse, CNRS, Université Paul Sabatier, 31062 Toulouse, France

**Keywords:** cancer cell clustering, anchorage-independent aggregation, transcriptional expression, Connectivity Map, HDAC inhibitors

## Abstract

**Simple Summary:**

Metastases formation involves the formation, circulation and seeding of cohesive group of tumor cells called circulating tumors cells clusters at distant organs from the primary tumor. These clusters have a much higher metastatic potential than individual circulating tumor cell, it is therefore important to understand the molecular mechanisms involved in their formation. To this aim, in this study, from the analysis of the relationship between in vitro aggregation quantitative characterization of 25 cancer cell lines and their expression data, we identified genes significantly associated with aggregation. Interestingly, we found that these genes were strongly correlated with the transcriptional signature induced by HDAC inhibitors treatment and we finally showed experimentally that two HDAC inhibitors inhibits tumor cells cluster formation in vitro. These results open new therapeutic perspectives to prevent metastasis formation.

**Abstract:**

Characterization of the molecular mechanisms involved in tumor cell clustering could open the way to new therapeutic strategies. Towards this aim, we used an in vitro quantitative procedure to monitor the anchorage-independent cell aggregation kinetics in a panel of 25 cancer cell lines. The analysis of the relationship between selected aggregation dynamic parameters and the gene expression data for these cell lines from the CCLE database allowed identifying genes with expression significantly associated with aggregation parameter variations. Comparison of these transcripts with the perturbagen signatures from the Connectivity Map resource highlighted that they were strongly correlated with the transcriptional signature of most histone deacetylase (HDAC) inhibitors. Experimental evaluation of two HDAC inhibitors (SAHA and ISOX) showed that they inhibited the initial step of in vitro tumor cell aggregation. This validates our findings and reinforces the potential interest of HDCA inhibitors to prevent metastasis spreading.

## 1. Introduction

The detection and monitoring of circulating tumor cells (CTC) have been the subject of major interest for several years to study and understand cancer pathology and to monitor the therapeutic response [1,2]. Indeed, tumor cell escape from the primary site and migration in the bloodstream to a secondary site contribute to metastatic disease [3]. Recent work has shown the existence of CTC clusters that are groups of cohesive cancer cells derived from the primary tumor [4] with a much higher metastatic potential than isolated CTCs. Furthermore, CTC association with neutrophils drives cell cycle progression, thus enhancing their metastatic potential [5].

Cell–cell adhesion involves multiprotein complexes located in intercellular junctions. In vertebrates, these cellular structures mostly comprise adherens junctions, gap junctions, desmosomes, and tight junctions. Proteins of the cadherin family are involved in adherens junctions (E-cadherin) and desmosomes (desmoglein and desmocolin); occludin, claudin, and ZO proteins are the most important components of tight junctions and Gap junctions connect two adjacent cells by establishing the continuity of connexons formed by connexins.

Several years ago, it was shown that increased expression of E-cadherin, a major component of cell adherens junctions, enhances the formation of cell aggregates [6]. This is consistent with the finding that loss of functional E-cadherin is associated with cancer cell invasiveness and metastasis formation [7]. On the other hand, increased cancer cell adhesion properties have also been associated with higher experimental metastatic potential in vivo [8,9]. In the same way, claudins expression is often decrease in cancers, but these proteins have also been implicated in the metastatic cascade [10]. It has been reported that the expression of plakoglobin, a major actor of desmosomes, is heterogeneous in the primary tumor [4], and that cells strongly expressing plakoglobin are at the origin of CTC cluster formation, which in turn promotes metastasis formation. In mouse models, plakoglobin knockdown abrogates CTC cluster formation and suppresses lung metastases [4]. Furthermore, in patients with breast or prostate cancer, overexpression of the JUP gene that encodes plakoglobin has been associated with poor prognosis [4]. Other cell adhesion molecules, such as CD44 or ICAM1, have also been shown to be involved in the clustering of cancer cells [11].

To identify additional molecular actors involved in the formation and/or stability of cancer cell clusters, study of the modalities and regulation of clustering also relies on in vitro assays to monitor intercellular interactions. We previously developed such an assay that we used to demonstrate the key positive role of E-cadherin and of two desmosomal proteins (DSG2 and DSC2), which interact with plakoglobin at this junction, in cancer cell aggregation [12]. We also showed the importance of gap junctions in the early stages of tumor cell aggregation [13]. Moreover, recently, we found that mitotic arrest prevents cancer cell clustering, and therefore, could reduce CTC metastatic potential upon treatment with microtubule-targeting anticancer drugs [14].

The characterization of the molecular mechanisms involved in tumor cell clustering could open the way to new therapeutic perspectives. Therefore, it is essential to identify the regulators of this process and their specific roles. With this aim in mind, we chose an approach based on a basic observation. Specifically, among tumor cell lines of epithelial origin, some display very high anchorage-independent clustering capacities in vitro and aggregate very rapidly, while others have very limited, if any, clustering capacity. Therefore, we analyzed the anchorage-independent aggregation capacity of 25 tumor epithelial cell lines. Then, we selected three quantitative aggregation dynamic parameters to evaluate their relationship with gene expression data for these cell lines extracted from the CCLE database [15]. This approach led to the identification of genes the expression of which was associated with these aggregation parameter variations. Comparison of these expression data with the perturbagen signatures in the Connectivity Map resource [16] highlighted a very high correlation with the signature of most histone deacetylase (HDAC) inhibitors. Finally, we experimentally confirmed this finding by demonstrating in vitro the inhibitory effect of the SAHA (vorinostat) and ISOX HDAC inhibitors on the early steps of MCF-7 cell aggregation.

Altogether, our observations support and strengthen the idea that deciphering the mechanism of tumor cell clustering and identifying the underlying regulatory pathways and interfering compounds might lead to new therapeutic opportunities.

## 2. Materials and Methods

### 2.1. Cell Culture

The 25 cancer cell lines (Table S1) were cultured in a humidified atmosphere of 5% CO_2_ at 37 °C in DMEM (Gibco, Life Technologies, Indianapolis, IN, USA), without or with 20 IU/L insulin (Sigma, St Louis, MO, USA), or in RPMI1640 (Gibco, Life Technologies) medium according to the supplier’s recommendations, both supplemented with 10% fetal calf serum (Gibco, Life Technologies) and 1% penicillin/streptomycin (100 U/mL, Gibco, Life Technologies). To study the impact of SAHA and ISOX, cells were incubated in culture medium containing 5 µM ISOX for 8 h or 10 µM SAHA for 24 h, then trypsinized and seeded for aggregation assay in culture medium containing the same concentration of ISOX and SAHA.

### 2.2. Cell Clustering Assay

The cell clustering assay used in this study was adapted with slight modifications from the one described in our previous reports [12,13]. Specifically, DMEM-F12 (Gibco Life Technologies) supplemented with 10% fetal calf serum (FCS) (Gibco Life Technologies) and 1% penicillin/streptomycin (100 U/mL, Gibco Life Technologies) was used as medium for all the cell lines. Cells (500 cells/well) were seeded in low-attachment round-bottomed 96-well plates (Costar^®^, Corning, New York, NY, USA), except in the 36 peripheral wells to avoid edge effects. Plates were centrifuged at 400 g for 4 min, and then cell aggregation in each well was followed by time-lapse video-microscopy.

### 2.3. Time-Lapse Microscopy

Based on our previous study [12,13], images were acquired with an inverted wide-field Zeiss Z1 Observer Axio microscope, using a 0.3 NA 10X objective and a CoolSNAP CDD camera (Roper scientific, Sarasota, FL, USA) or an Axiocam506 (Zeiss, Oberkoshen, Germany) in bright field (transmitted light) for 6 to 24 h with one acquisition every 15 min. At each time point and for each well, 20-µm spaced z-stacks over 160 µm depth (9 stacks) in bright field were acquired using the MetaMorph or Zen blue software.

### 2.4. Quantification of Cell Clustering Parameters

The segmentation algorithm used to quantify the parameters of each tumor cell cluster is structured as follows:

- For each position/well/time, the whole stack of 2D images is fused into one single in-focus image. This process is based on a Laplacian of Gaussian filter to detect the in-focus image regions, followed by a Gaussian blending of these regions taken from the different focal planes.
- The background signal of the resulting in-focus image is estimated using a morphological opening and then subtracted.
- A Gaussian smoothing filter is then applied and the image intensities adjusted (histogram equalization performed on the pixel intensity to obtain a saturation of 1% of the extreme values), followed by a simple automated thresholding, resulting in a binary mask that corresponds to all cell aggregates.
- Cell aggregates are individually detected in the binary image as connected components that match several criteria, and are divided in two groups according to their area using a threshold of 10,000 pixels (or 4160 µm^2^, because the XY resolution is 0.645 µm at 10× magnification). Detected objects with an area smaller than this threshold are excluded from the analysis. Holes inside the binary objects are excluded in the two object categories to measure standard parameters: number of aggregates, normalized area to the initial time point, and circularity. This image analysis pipeline was developed within a parallelized processing architecture using multi-core processors.

**Figure 1:**
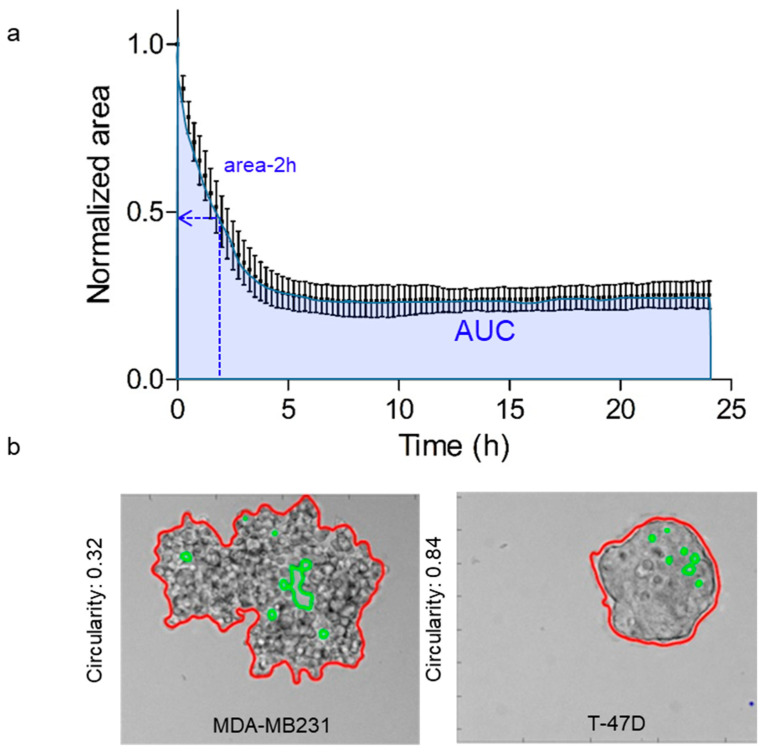
Determination of the quantitative parameters of anchorage-independent cancer cell aggregation dynamics. (**a**) Definition of the parameters extracted from the average aggregation curves of the normalized area. Each value of the normalized area, measured every 15 min, is the mean of 3–5 independent experiments with error bars. The area-2h is the area at 2 h and AUC is the area under the curve. (**b**) Micrographs: determination of the aggregate circularity after 24 h using an automated image analysis procedure. The red line shows the contours of the aggregate and the green color marks areas without cells. MDA-MB-231 (left) and T-47D (right) cells are shown as examples of circularity variations after 24 h. The circularity values are indicated on the side of the micrographs.

For this study, from the curve representing the changes of the normalized area during cell aggregation (Figure 1), two parameters were extracted: the projected aggregate area at 2 h (area-2h) to take into account the early dynamics of the aggregation process and the Area Under the Curve (AUC) to consider the whole aggregation process. Additionally, to include a parameter that describes the degree of compaction of the aggregates, the aggregate circularity was determined on the basis of automated image segmentation analysis using the following equation: Circularity = 4 × *pi* (Area/Perimeter^2^); a circularity of 1 indicates a perfect circle (Figure 1).

### 2.5. Analysis of the Relation between Cell Aggregation Parameters and CCLE Transcriptional Data

The gene expression data for the 25 cell lines used in this study were downloaded from the CCLE website (https://portals.broadinstitute.org/ccle/ accessed on: 4 June 2019). [15,17]. A sparse PLS method [18] was used to unravel the relationship between the three chosen aggregation parameters (AUC, area-2h, and circularity) and the gene expression. The PLS method relies on the maximization of the covariance between a combination of variables in two datasets (here, cell aggregation parameters and gene expression data). The sparse extension of the PLS, based on a LASSO penalty [19], allowed selecting the genes most associated with the aggregation parameters. This analysis was done with the mixOmics package [20] of the R software Version 1.2.5033 [21].

### 2.6. Connectivity Map Analysis

The Connectivity Map (CMap) is a resource that uses cell responses to perturbation to find relationships between diseases, genes, and therapeutics. The CMap database contains over one million gene expression signatures upon treatment of a variety of cell types with many different perturbagens (from small-molecule compounds to gene overexpression and gene knockdown reagents). This resource has been successfully used in various studies in the field of cancer research to identify new candidates based on original signatures and to propose new drug repurposing opportunities (see for instance [22,23,24,25]). Changes in gene expression that arise from treatment with nearly 2500 perturbagens were compared with the expression signature deduced from our correlation analysis to find similarities. The observed connectivity score (tau), which is a standardized measure ranging from −100 to 100, was recorded for each perturbagen. For more details on the connectivity score calculation and significance, see the information on this resource at https://clue.io/connectopedia (accessed on: 31 August 2020).

### 2.7. Statistical Analysis

Statistical analyses were performed with the GraphPad Prism software.

## 3. Results

### 3.1. Investigation of the Aggregation Parameters in a Panel of 25 Cancer Cell Lines

We previously showed that cancer cells seeded in anchorage-independent conditions spontaneously aggregate and form spherical clusters. The experimental assay we used here to determine the aggregation parameters consisted of monitoring by video-microscopy the kinetics of cancer cell cluster formation in low-attachment 96-well plates (Figure S1A,B). We quantified the aggregation dynamic parameters by measuring the average projected area of the multiple replicate aggregates relative to the area at time 0 from the automated segmentation of the microscopy recording (Figure S1C) [12,13].

**Figure 2:**
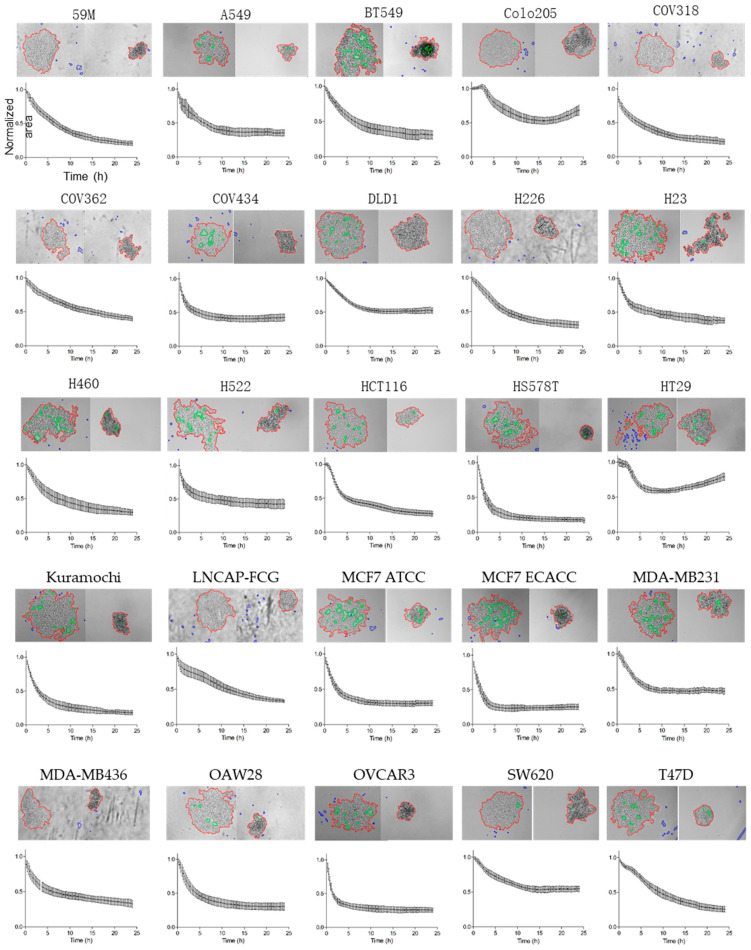
Anchorage-independent aggregation of 25 cancer cell lines. For each cell line, micrographs at time 0 (left) and after 24 h of aggregation (right) (top panel) and the curve of the normalized area (mean values from 28 to 48 replicates and three to five independent experiments) (lower panel) are shown.

In this study, we used this assay to test 25 cancer cell lines of various origins (Table S1). As illustrated by the micrographs taken at time 0 (initiation of cell aggregation) and at 24 h (Figure 2), cell aggregation was heterogeneous in the tested cancer cell lines, and did not always result in the formation of circular aggregates that will form a compact structure. This difference in aggregation capacity among cancer cell lines was also reflected in the curves representing the decrease in the relative area during the aggregation test (Figure 2). Moreover, these curves (obtained from 25 to 48 replicates for each cell line using data from three to five independent experiments) confirmed that this variability was not the result of a single observation, but of the intrinsic properties of each cell line.

Noteworthy, the two MCF7 cell lines from two different suppliers (ATCC and ECACC) behaved very similarly in this assay.

Our aim was to analyze the relationship between this variability and the gene expression profiles of the tested cell lines to identify new regulators of tumor cell clustering. Thus, to compare all 25 cell lines, we extracted three quantitative parameters that precisely described the aggregation kinetics and characteristics: the area-2h (early aggregation dynamics), the AUC (whole aggregation process), and circularity. These values are presented in Table 1.

### 3.2. Identification of Genes Associated with the Aggregation Quantitative Parameters

To identify genes the expression of which was associated with the aggregation quantitative parameters, we extracted the expression data of 19,851 genes for the 25 cell lines used in our study from the Cancer Cell Line Encyclopedia (CCLE) project (www.broadinstitute.org/ccle accessed on: 4 June 2019), a collaboration between the Broad Institute and Novartis [15,17]. Then, using the sparse PLS method, we identified a list of genes with transcript levels positively or negatively associated with variations of the AUC and area-2h (positive = 99; negative = 201) and of circularity (positive = 107; negative = 193) (Table S2).

### 3.3. Connectivity Map Analysis Identifies Strong Relationship with HDAC Inhibitors

**Figure 3:**
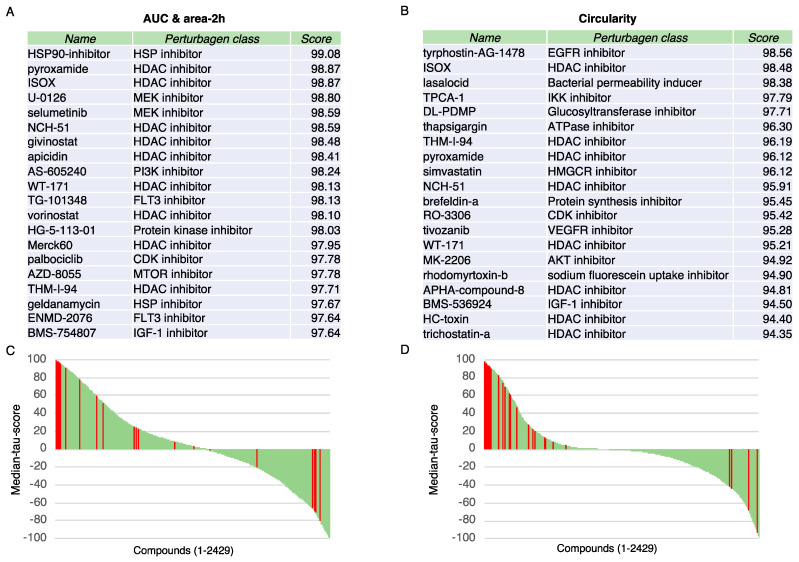
Connectivity Map analysis identifies HDAC inhibitors. (**A**,**B**) Top twenty compounds, the gene expression signature of which displays a connectivity score above ≥90 with the gene expression signatures for AUC and area-2h (**A**) and Circularity (**B**). (**C**,**D**) Connectivity score distribution for the gene expression signatures for AUC and area-2h (**C**) and Circularity (**D**) and all the 2429 perturbagen signatures (green bars). The results are expressed as median tau scores, with a scale-free measure ranging from −100 to 100. HDAC inhibitors are indicated with red bars.

As the genes associated with area-2h and AUC were different from those associated with circularity, we could not identify putative regulators of tumor cell aggregation directly with the sparse PLS method. Therefore, we used the CMap resource [16] to compare the identified gene (Table S2) with the transcriptional signatures obtained after treatment of a variety of cell types with small-molecule compounds (*n* = 2429 perturbagens). The complete list of the median tau scores for the perturbagens is provided as Supplementary Materials (Table S3), and the perturbagens with the highest 20 top connectivity scores are listed in Figure 3 (top panels). Among the hundred molecules with the highest scores, HDAC inhibitors were the most represented inhibitor class in the analyses carried out with the genes associated with area-2h and AUC (Figure S2A) and with circularity (Figure S2B) (Figure 3, lower panels).

These results suggest that HDAC inhibitors could regulate the capacity of cancer cells to form clusters.

### 3.4. Effect of HDAC Inhibitors on In Vitro Cancer Cell Aggregation

These results and a recent publication showing that HDAC inhibitors reduce CTC cluster size [26] led us to examine the effect of HDAC inhibition on cancer cell aggregation dynamics. Therefore, we incubated MCF-7 cells, which displayed rapid and efficient aggregation kinetics, leading to the formation of cohesive and circular aggregates (Figure 2 and Table 1), with SAHA (also known as vorinostat) and ISOX, two of the HDAC inhibitors identified as top hits from the Connectivity Map signature analysis.

**Figure 4:**
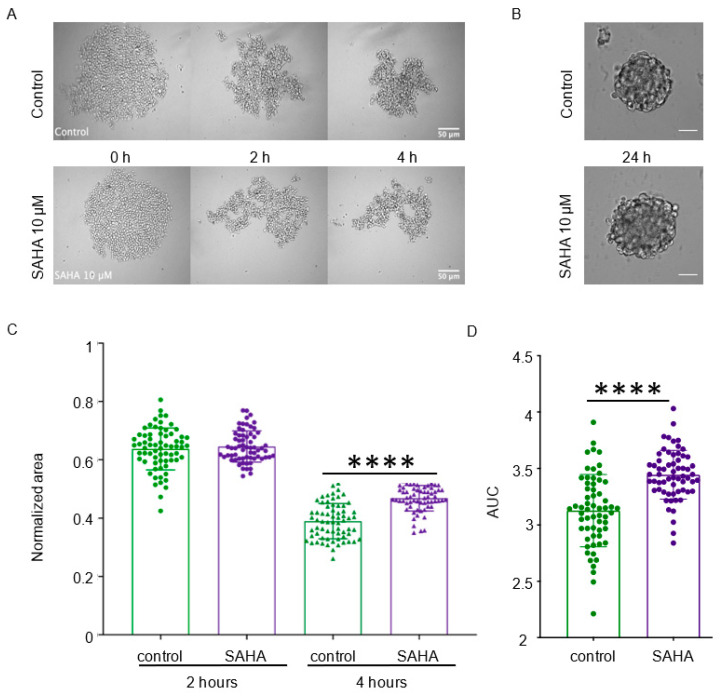
The HDAC inhibitor vorinostat impairs the early steps of cell clustering. (**A**) MCF-7 cells were incubated with 10 µM of SAHA (vorinostat) or not (control) for 24 h and then their aggregation capacity was tested. Micrographs at the indicated time points during the aggregation assay are shown. (**B**) Micrographs of the aggregates formed after 24 h of incubation with or without SAHA. (**C**) The normalized area of cell aggregates in the presence or not of SAHA (10 µM) was measured at 2 h and 4 h after initiation of the aggregation assay. Each dot corresponds to one replicate and bars are the mean ± SD (*n* = 4 independent experiments). (**D**) AUC of cell aggregates in the presence or not of SAHA (10 µM) after 6 h of aggregation. Data are the mean ± SD. Control *n* = 61 aggregates, SAHA *n* = 62 from four independent experiments. ****, *p* < 0.0001 (Mann–Whitney non-parametric test).

In the presence of SAHA, cells progressively aggregated (Figure 4A), without any obvious effect on spheroid formation at 24 h after seeding (Figure 4B). No cell death was visualized in the experimental condition used (data not shown). However, analysis of the relative area after 2 h and 4 h of aggregation and of the AUC (Figure 4C,D) from the aggregation curves showed that after 4 h, the relative area (mean value: 0.39 ± 0.06 vs. 0.47 ± 0.04; *p* < 0.0001) and the AUC (mean value: 3.12 ± 0.32 vs. 3.44 ± 0.21; *p* < 0.0001) were significantly higher in SAHA-treated cells than controls. We obtained similar results in cells incubated with ISOX (Figure S3). This indicates that the HDAC inhibitors SAHA and ISOX slightly, but significantly, inhibit the early steps of tumor cell aggregation, suggesting that these compounds could affect the cancer cell capacity to form clusters.

## 4. Discussion

It is acknowledged that CTC clusters have a much higher metastatic potential than isolated CTCs [4]. This finding justifies the importance of identifying the molecular and cellular mechanisms that induce or reinforce tumor cell cohesion after their escape from the primary tumor and during their circulation in the bloodstream. We addressed this question in vitro by developing assays to explore at the cell population scale [12] and in isolated cells [14] the aggregation capacities of tumor cells in the absence of anchorage. This strategy allowed us to identify by a candidate approach the role of the desmosome components DSC2 and DSG2, of E-cadherin, and of gap junctions in this process [12,13]. Interestingly, plakoglobin, which is also a desmosomal protein has been shown has been shown to be overexpressed in CTC clusters compared to single CTCs and in mouse models, plakoglobin knockdown abrogates CTC cluster formation and suppresses lung metastases. Thus, the results obtained on cancer cell lines can be related to the data on CTCs. Using this in vitro assay, here, we compared the anchorage-independent aggregation features of 25 tumor lines, and then used the obtained data to identify new regulators. For the aggregation capacity comparison, we made the arbitrary choice to use the same experimental conditions for all cell lines (i.e., assay performed in the same culture medium). We tested all cell lines in three to five independent experiments and we quantified the aggregation kinetic parameters using data from 25–48 replicates to obtain robust results with low experimental variability (see Figure 2).

Our results show a strong aggregation parameter heterogeneity among cell lines, without any obvious correlation between the obtained results and the tissue of origin of the tumor. Importantly, we obtained similar results in the two MCF-7 mammary adenocarcinoma cell lines from two different suppliers (ECACC and ATCC), which confirms the reliability of the methodology used.

We focused on three quantitative parameters: area-2h, AUC, and circularity. First, we identified genes that showed expression variations in the 25 cell lines associated with these aggregation parameters using data from the CCLE transcriptional database [15,17]. Then, we performed a connectivity analysis using these genes and the transcriptional signatures of 2429 perturbagens from the CMap resource database [16]. In this analysis, a large number of HDAC inhibitors were among the first hits with the highest connectivity scores. Finally, we tested the impact of two HDAC inhibitors with very high connectivity scores (vorinostat (SAHA) and ISOX)) on MCF-7 cell aggregation in vitro. This analysis showed that these two HDAC inhibitors have a significant inhibitory effect mostly on the first part (T = 4 hours) of the aggregation process. At later time points, this inhibitory effect disappeared, probably compensated by the other mechanisms that control intercellular interactions, similarly to what we observed with gap junction inhibitors [13].

A recent comparative analysis [26] demonstrated that in patients with breast cancer, a hypomethylation signature (OCT4, SOX2, NANOG and SIN3A) in CTC clusters is associated with poor progression-free survival. Moreover, the screening of 2486 FDA-approved compounds for their ability to dissociate clusters of patient-derived CTCs identified 39 candidates, including two HDAC inhibitors (vorinostat and pracinostat). Pharmacological dissociation of CTC clusters led to remodeling of the methylation profile and suppressed metastasis formation. These observations strongly support the relevance and interest of our own results. An attractive hypothesis would be that histone acetylation is associated with decreased tumor cell clustering capacity and that HDAC are positive regulators of this process, which would then reinforce the interest of HDCA inhibitors to prevent metastasis spreading. Histone deacetylases (HDAC) could regulate cancer cell aggregation through regulation of cell junction proteins. Indeed, it has recently been shown that HDACs regulate the expression of Claudin-1 and Claudin-2 in colon cancer cells [27,28]. Additional in vitro and in vivo studies are required to validate these hypotheses.

## Figures and Tables

**Table 1:** Summary of the three aggregation parameters (area-2h, AUC, and circularity) extracted from the aggregation curves of the 25 cancer cell lines under study. Values are the mean of 25 to 48 replicates from three to five independent experiments.

	Area-2h	AUC	Circularity
**59M**	0.774	9.853	0.6451
**A549**	0.724	10.88	0.4763
**BT549**	0.786	10.97	0.4133
**COLO205**	1.03	16.3	0.5596
**COV318**	0.646	9.018	0.6619
**COV362**	0.838	14.01	0.4843
**COV434**	0.596	11.04	0.5506
**DLD1**	0.85	14.26	0.6126
**H226**	0.86	11.66	0.4747
**H23**	0.693	11.26	0.3673
**H460**	0.772	11.13	0.4898
**H522**	0.644	11.99	0.4031
**HCT116**	0.782	10.39	0.6901
**HS578T**	0.477	6.277	0.8117
**HT29**	0.966	16.76	0.4627
**KURAMOCHI**	0.581	7.287	0.5922
**LNCAP**	0.779	12.76	0.6773
**MCF7 ATCC**	0.584	8.887	0.5947
**MCF7 ECACC**	0.47	6.93	0.5506
**MDAMB231**	0.83	13.25	0.3204
**MDAMB436**	0.675	11.14	0.4921
**OAW28**	0.68	9.755	0.3956
**OVCAR 3**	0.433	7.55	0.5445
**SW620**	0.873	15.17	0.3807
**T47D**	0.854	11.76	0.8374

## Data Availability

The data presented in this study are available in the figures and in the supplementary materials.

## Supplementary Materials

The following are available online at https://www.mdpi.com/article/10.3390/cancers13225840/s1, Figure S1. Procedure to evaluate anchorage-independent aggregation of cancer cells. Figure S2: Distribution of perturbagens with the 100 top scores according to their class. Figure S3. ISOX impairs the early steps of cell clustering. Table S1. List of the cell lines used in this study. Table S2. List of genes with transcript level significantly positively or negatively associated with the AUC and area-2h or with Circularity (Excel file). Table S3. Connectivity Map analysis. Complete list of perturbagens and their connectivity scores (absolute values) with the gene expression signatures for AUC and area-2h or Circularity. HDAC inhibitors are highlighted in yellow (Excel file).
