# Supplementary material for: Quantitative Analysis of Cell Aggregation Dynamics Identifies HDAC Inhibitors as Potential Regulators of Cancer Cell Clustering
Source: Cancers (Basel). 2021 Nov 21;13(22):5840. doi: 10.3390/cancers13225840 (PMC8616495; doi:10.3390/cancers13225840)
Supplement: Supplementary file 1 [file cancers-13-05840-s001.zip › cancers-1433631-supplementary/FigS1.pdf]

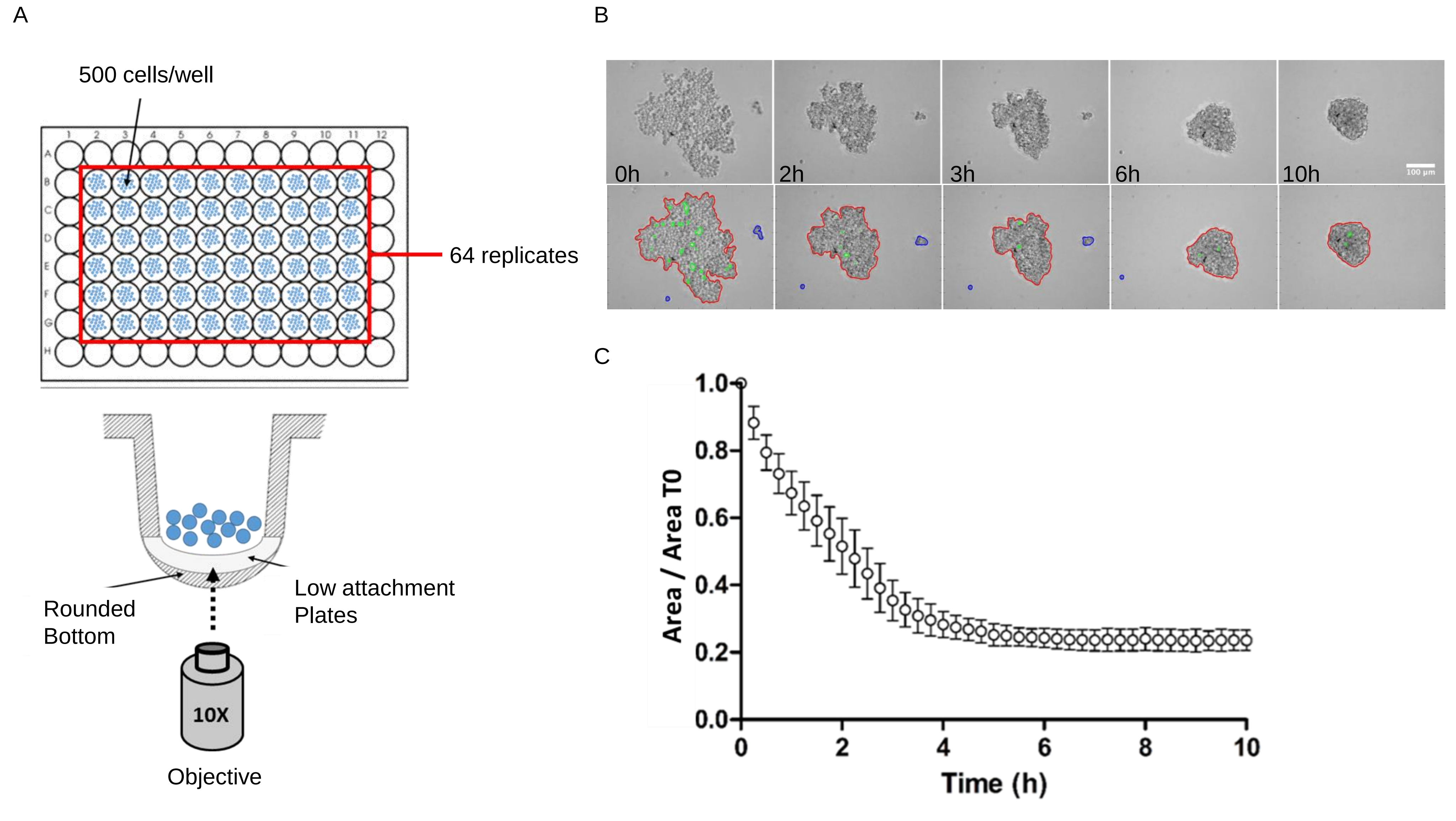

Supplementary Figure S1. Procedure to evaluate anchorage-independent aggregation of cancer cells.

(A) Schematic of the procedure. To avoid edge effects, only 64 samples and/or replicates are seeded and analyzed in each experiment. 500 cells are seeded in each well of a round bottom low-attachment plate. A short centrifugation at 400g allows gathering the cells together at time 0. (B) Representative microscopy images of MCF-7 cell aggregation at the indicated time points. Segmentation (red line) was performed using a dedicated MATLAB software. Green color indicates the excluded holes, and blue lines isolated cells. (C) Representative curve of the normalized area (Area/Area at time 0) obtained with 41 replicates from 5 independent experiments (mean ± SD).
