# Supplementary material for: Quantitative Analysis of Cell Aggregation Dynamics Identifies HDAC Inhibitors as Potential Regulators of Cancer Cell Clustering
Source: Cancers (Basel). 2021 Nov 21;13(22):5840. doi: 10.3390/cancers13225840 (PMC8616495; doi:10.3390/cancers13225840)
Supplement: Supplementary file 1 [file cancers-13-05840-s001.zip › cancers-1433631-supplementary/FigS2.pdf]

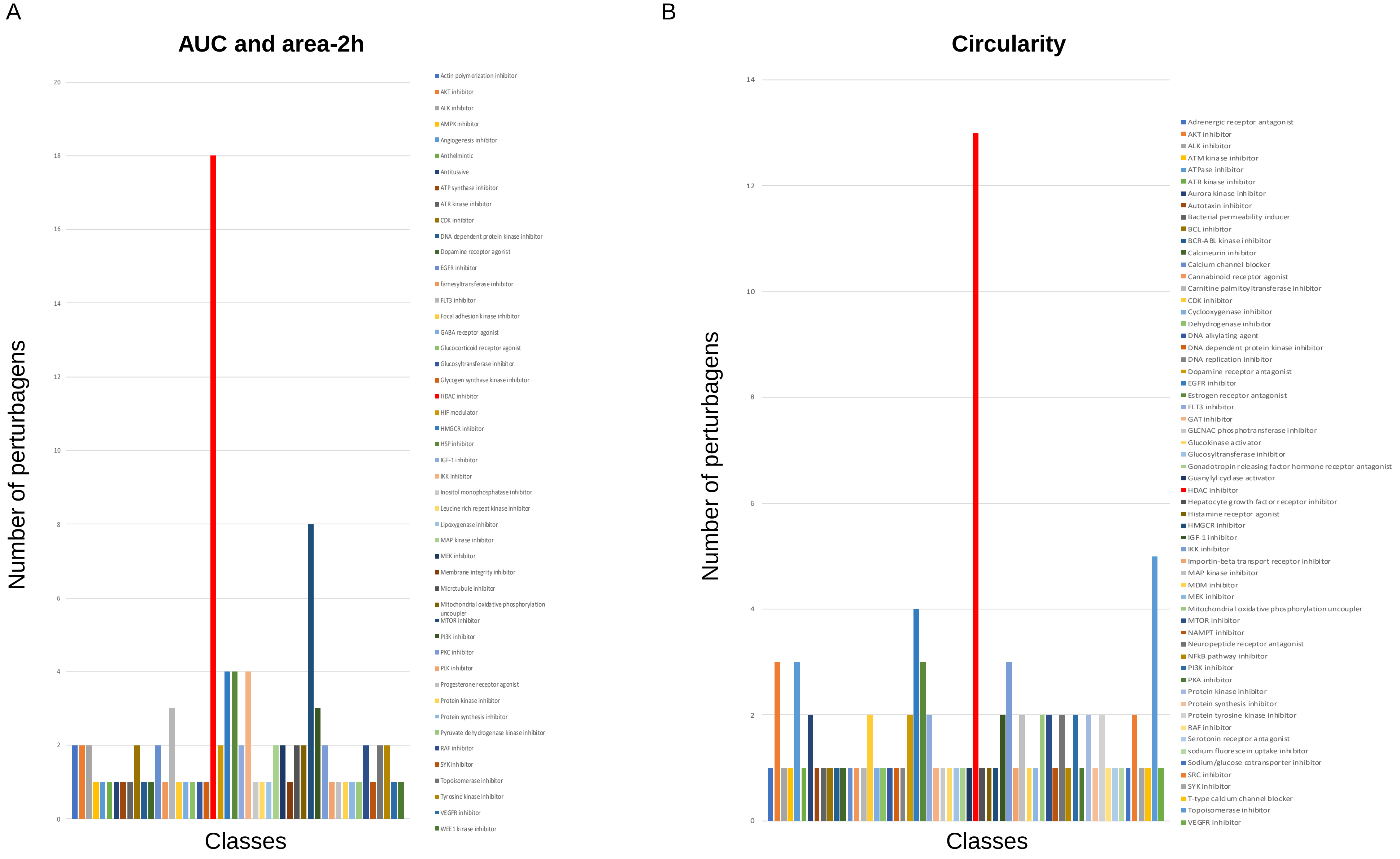

Supplementary Figure S2: Distribution of perturbagens with the 100 top scores according to their class.

Each graph corresponds to the number of compounds, with the 100 top scores, in each indicated class for the results obtained with the gene expression signatures for AUC and area-2h (A) and for circularity (B). In both cases, HDCA inhibitors (red) are the most represented class.
