# Supplementary material for: Quantitative Analysis of Cell Aggregation Dynamics Identifies HDAC Inhibitors as Potential Regulators of Cancer Cell Clustering
Source: Cancers (Basel). 2021 Nov 21;13(22):5840. doi: 10.3390/cancers13225840 (PMC8616495; doi:10.3390/cancers13225840)
Supplement: Supplementary file 1 [file cancers-13-05840-s001.zip › cancers-1433631-supplementary/FigS3.pdf]

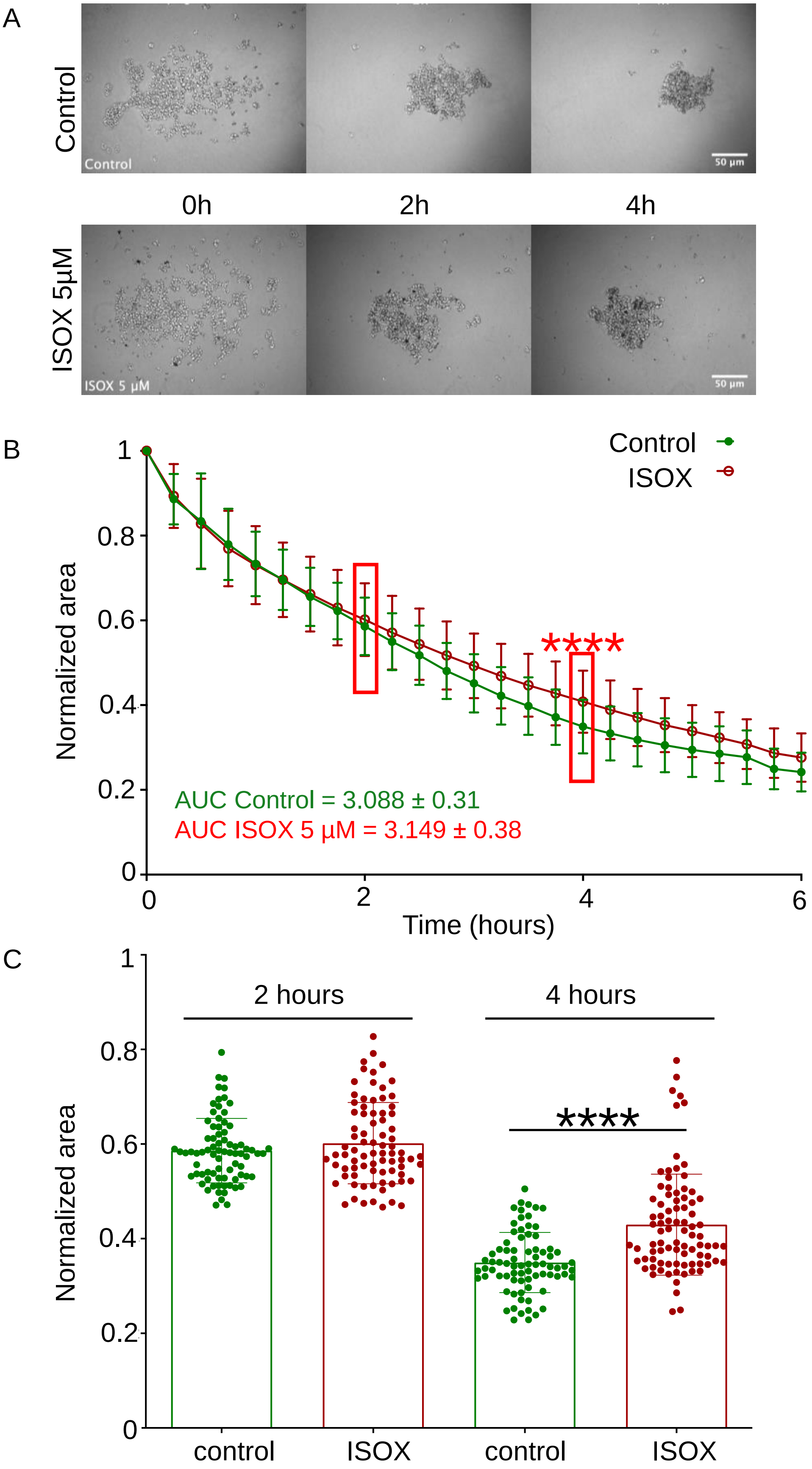

Supplementary Figure S3. ISOX impairs the early steps of cell clustering.

(A) Aggregation assay (T=0, 2 hours and 4 hours) in MCF-7 cells in the presence or not (control) of the HDCA inhibitor ISOX (5µM) and after 8 hours of pre-incubation or not (control). (B) Normalized area of cell aggregates in the presence or not of ISOX and after a pre-incubation or not of 8 hours. Data are the mean ± SD. AUC is the Area Under Curve. (C) Normalized area of cell aggregates in the presence or not of ISOX (5µM) after 2 hours and 4 hours of aggregation. Data are the mean ± SD from six independent experiments. Control n=78 aggregates, ISOX n=80 aggregates. ****, P <0.0001 (Mann Whitney non-parametric test).
