# Supplementary material for: Quantitative Analysis of Cell Aggregation Dynamics Identifies HDAC Inhibitors as Potential Regulators of Cancer Cell Clustering
Source: Cancers (Basel). 2021 Nov 21;13(22):5840. doi: 10.3390/cancers13225840 (PMC8616495; doi:10.3390/cancers13225840)
Supplement: Supplementary file 1 [file cancers-13-05840-s001.zip › cancers-1433631-supplementary/TableS1.pdf]

Cell lines	Origin	Source	Culture media
59M	Ovarian	ECACC	DMEM, 20IU/l insulin
COV318	Ovarian	ECACC	DMEM
COV362	Ovarian	ECACC	DMEM
COV434	Ovarian	ECACC	DMEM
KURAMOCHI	Ovarian	JCRB cell bank	RPMI1640
OAW28	Ovarian	ECACC	DMEM, 20IU/l insulin
OVCAR3	Ovarian	ATCC	RPMI1640
A549	Lung	ATCC	RPMI1640
H23	Lung	ATCC	RPMI1640
H226	Lung	ATCC	RPMI1640
H460	Lung	ATCC	RPMI1640
H522	Lung	ATCC	RPMI1640
COLO205	Colon	ATCC	RPMI1640
DLD1	Colon	ATCC	RPMI1640
HCT116	Colon	ATCC	DMEM
HT29	Colon	ATCC	DMEM
SW620	Colon	ATCC	RPMI1640
BT-549	Breast	ATCC	RPMI1640
HS-578T	Breast	ATCC	DMEM
MCF-7 ATCC	Breast	ATCC	RPMI1640
MCF-7 ECACC	Breast	ECACC	DMEM
MDA-MB-231	Breast	ATCC	DMEM
MDA-MB-436	Breast	ATCC	DMEM
T47-D	Breast	ATCC	RPMI1640
LNCaP-FCG	Prostate	ATCC	RPMI1640

Supplementary Table S1. List of the cell lines used in this study

The name of the 25 cell lines, tissue of origin, their commercial source, and culture medium are indicated.
